# 1.5 and 3 Tesla quantification of myocardial perfusion reserve in comparison to fractional flow reserve

**DOI:** 10.1186/1532-429X-15-S1-P199

**Published:** 2013-01-30

**Authors:** Peter Bernhardt, Thomas Walcher, Wolfgang Rottbauer, Jochen Wöhrle

**Affiliations:** 1 University of Ulm, Ulm, Germany

## Background

Quantitative myocardial perfusion reserve (MPR) analysis is capable of noninvasive detection of significant coronary artery disease. However, little is known about MPR evaluation in comparison to fractional flow reserve (FFR), especially at 3 Tesla. Aim of our study was to compare quantitative MPR at 1.5 and 3 Tesla against FFR.

## Methods

Thirty-four patients referred for coronary x-ray angiography with suspected or known coronary artery disease underwent cardiac magnetic resonance imaging (CMR) at 1.5 and 3 Tesla, and additionally FFR measurement in LAD, CX and RCA. MPR was calculated in 544 myocardial segments at both field strenghts. FFR was measured in 109 coronary arteries with a cut-off value of ≤0.8 for relevant stenosis.

## Results

In 38 coronary arteries (N=19 LAD, N=8 CX, N=11 RCA) a reduced FFR ≤0.8 was found. Receiver operator curve analysis yielded a higher area under the curve for 3 Tesla in comparison to 1.5 Tesla MPR (0.96 vs 0.65, p<0.001) resulting in higher values for sensitivity (90.5% vs. 61.9%) and specificity (100% vs. 76.9%), respectively.

## Conclusions

Both field strengths, 1.5 and 3 Tesla, are capable to detect hemodynamic significant coronary artery stenoses using quantitative MPR analysis. The diagnostic accuracy at 3 Tesla is however superior to 1.5 Tesla for MPR quantification.

## Funding

The study was partially funded by a research grant of Guerbet (France).

